# Late-Onset Familial Primary Cardiac Angiosarcoma in Two Sisters

**DOI:** 10.1093/icvts/ivag114

**Published:** 2026-07-21

**Authors:** Hiroto Kawakami, Hiroshi Tsuneyoshi, Shoichi Kyo, Makoto Suzuki

**Affiliations:** Department of Cardiovascular Surgery, Shizuoka General Hospital, Shizuoka 420-8527, Japan; Department of Cardiovascular Surgery, Shizuoka General Hospital, Shizuoka 420-8527, Japan; Department of Cardiovascular Surgery, Shizuoka General Hospital, Shizuoka 420-8527, Japan; Department of Pathology, Shizuoka General Hospital, Shizuoka 420-8527, Japan

**Keywords:** cardiac tumour, primary cardiac angiosarcoma, heredity, familial cancer syndrome

## Abstract

**Introduction:** Primary cardiac angiosarcoma is an exceptionally rare malignancy. Familial occurrences are even less common and associated with hereditary cancer syndromes; notably, all previously reported familial cases occurred in young individuals.

**Case:** We encountered 2 elderly sisters who both developed morphologically similar primary cardiac angiosarcomas arising from the right atrium. The elder sister presented with nocturnal wheezing, and a large right atrial tumour was found at age 70. She underwent surgical resection followed by radiotherapy. The younger sister presented with a similar cough, and screening transthoracic echocardiography revealed a right atrial mass at age 74. She received similar treatment.

**Discussion:** These cases suggest a potential hereditary predisposition and further highlight that familial occurrence of primary cardiac angiosarcoma can manifest even at advanced age, challenging the conventional understanding of this disease.

## INTRODUCTION

Primary cardiac neoplasms are exceedingly rare, occurring in 0.0001%-0.030% of autopsy cases; approximately 25% are malignant, with angiosarcoma being the most common subtype.^1^ It typically develops in individuals younger than 65 years and predominantly arises in the right atrium (RA).^1^

Familial occurrence is even rarer and associated with hereditary cancer syndromes. In 4 previously reported familial cases,^2–4^ the onset was early, between the second and fifth decades.

Surgery is the mainstay of treatment, particularly for localized disease.^1^ The standard approach is tumour resection and reconstruction, with valve or coronary procedures as needed. For complex left-sided lesions, orthotopic autologous heart transplantation represents a therapeutic option. When tumour size or extent precludes functional reconstruction, heart transplantation or a total artificial heart may be considered. However, in Japan, these options are not available.

We report 2 sisters, in their seventies, who developed morphologically similar right atrial angiosarcomas and underwent surgical resection.

## CASE PRESENTATION

### Case 1

**Figure 1.**
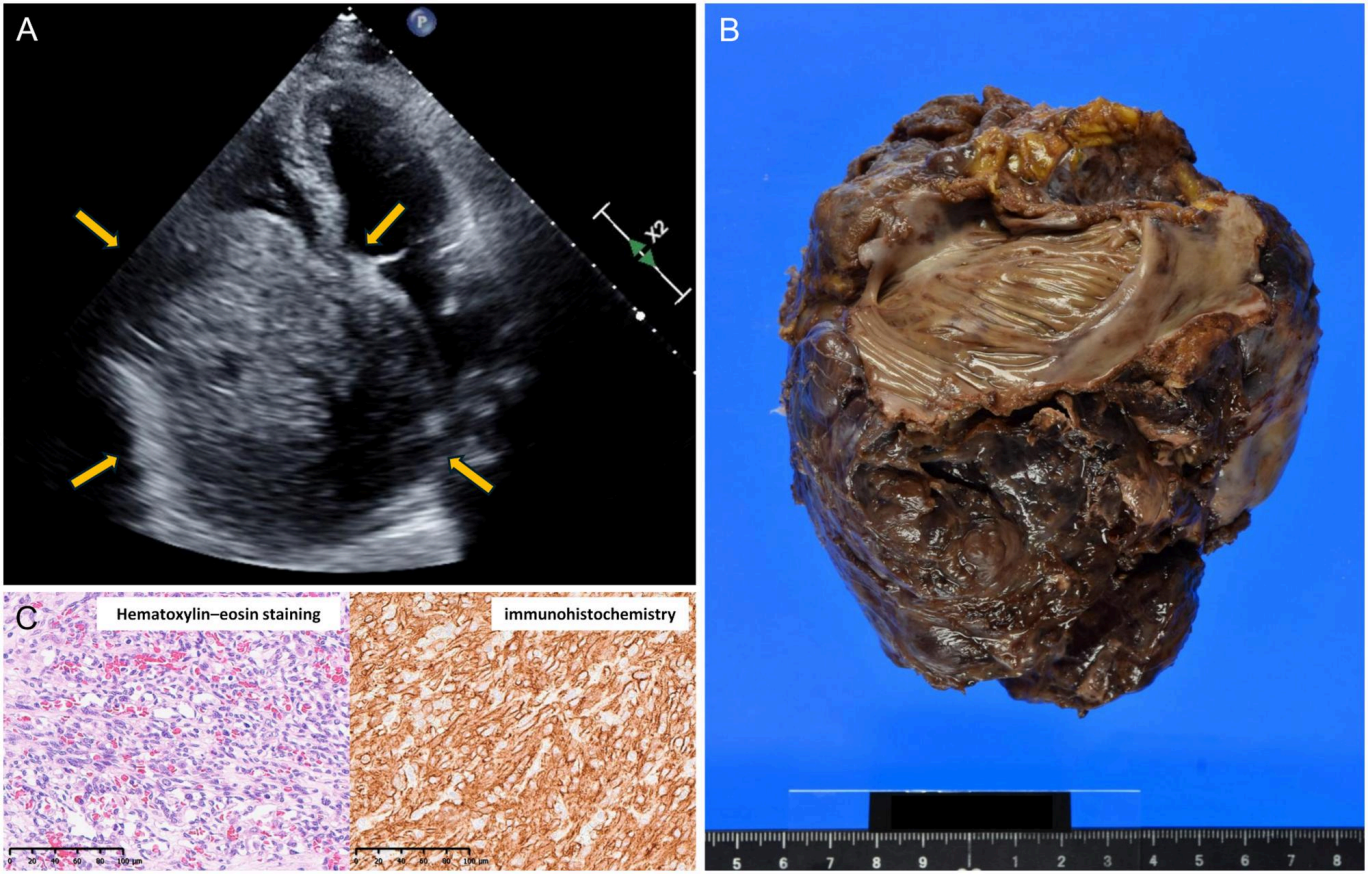
(A) Transthoracic Echocardiography Showing a Large Right Atrial Mass (Arrows). (B) Gross specimen of a 13 × 11.5 × 7 cm haemorrhagic tumour. (C) Haematoxylin–eosin staining: densely proliferating spindle cells forming slit-like vascular channels with necrosis. Immunohistochemistry: CD31, Factor VIII, α-SMA, and CD99 positive.

A 70-year-old woman presented with nocturnal wheezing. Her history included thyroid adenoma. Transthoracic echocardiography (TTE) (**Figure 1A**) and contrast-enhanced computed tomography (CT) (**Video 1**) revealed a large right atrial mass with no metastasis. Computed tomography-guided biopsy confirmed angiosarcoma.

Median sternotomy revealed tumour-filled, densely adherent pericardium. The RA was occupied by tumour extending to the inferior vena cava (IVC); cardiopulmonary bypass (CPB) was established with femoral artery inflow and femoral vein and superior vena cava (SVC) drainage. After arrest, the RA was opened from the tumour-free SVC. The tumour extended to the left atrial roof and involved the right coronary artery (RCA). It was resected with a 5-mm margin; the RCA was separable. Resection reached within 1 mm of the tricuspid annulus and extended to the diaphragmatic IVC. The defect was reconstructed with a bovine pericardial patch. After de-clamping, transoesophageal echocardiography showed moderate tricuspid regurgitation (TR). The tricuspid annuloplasty was performed after re-clamping, with sutures partially placed through the patch covering the tricuspid annulus. A 30 mm TriAd ring was implanted with a central clover stitch for residual TR.

Histopathology confirmed angiosarcoma (**Figure 1B and C**) with focally positive margins. She was discharged on postoperative day 14 and received radiotherapy. She developed multiple bone metastases and later intracerebral Haemorrhage of undetermined relation to the tumour, dying 8 months after surgery.

### Case 2

**Figure 2.**
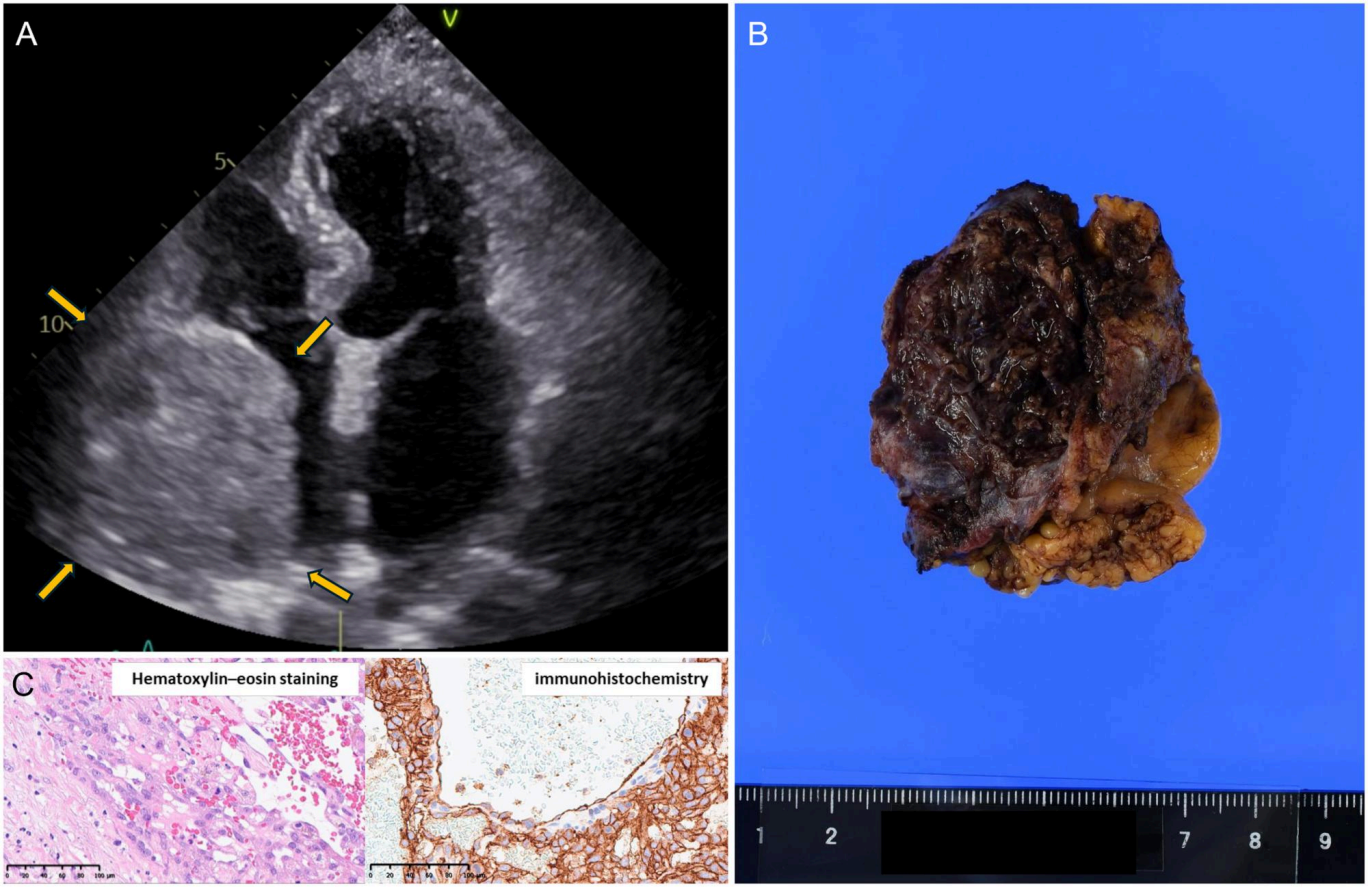
(A) Transthoracic Echocardiography Showing a Right Atrial Mass (Arrows). (B) Gross specimen of a 7.5 × 4.5 × 3.2 cm haemorrhagic tumour. (C) Haematoxylin–eosin staining: spindle-to-epithelioid cells forming irregular vascular channels and solid sheets with haemorrhage and necrosis. Immunohistochemistry: CD31, CD34, and Factor VIII positive.

A 73-year-old younger sister, the only sibling, presented with a similar cough. Her history included uterine leiomyoma. No other family history of malignancy existed. Screening TTE revealed only pericardial effusion. Serial TTE 1 year later identified a new right atrial mass (**Figure 2A**). Contrast-enhanced CT confirmed the tumour (**Video 2**) with no metastasis.

Median sternotomy revealed dense pericardial adhesions with bloody effusion and tumour invasion of the pericardium and right pleura. The inferior RA was replaced by tumour; CPB was established with ascending aortic inflow and SVC and FV drainage. On the beating heart, the tumour was resected with a 5-mm margin; the RCA was involved but dissectible along its entire length. Resection extended to the IVC orifice, spared the SVC, and preserved 1 cm from the tricuspid annulus. The defect was reconstructed with a bovine pericardial patch. All gross invasion of the pericardium, right pleura, and right lung was resected.

.Histopathology confirmed angiosarcoma (**Figure 2B and C**) with positive pleural margins. She was discharged on postoperative day 11 and received radiotherapy; however, disease progressed, and she died 3 months postoperatively.

## DISCUSSION

These cases highlight a crucial clinical finding. Familial occurrence of primary cardiac angiosarcoma can manifest even in elderly individuals. In the previous 4 cases,^2–4^ onsets occurred between the second and fifth decades. Familial occurrence is considered related to Li-Fraumeni syndrome with TP53 mutations or Li-Fraumeni-like families with germline POT1 mutations.^4^ In 3 of the 4^2–4^ cases, genetic testing was performed and identified hereditary pathogenic variants. Beyond familial cases, recent studies have identified several causative genes implicated in primary cardiac angiosarcoma, including germline POT1 mutations, recurrent MED12 alterations, and KDR variants.^5^

Genetic testing was not performed because the patients and their family declined the procedure. Although the late onset may be consistent with sporadic disease, the rarity of primary cardiac angiosarcoma and the near-synchronous occurrence in 2 first-degree relatives suggest a shared hereditary predisposition.

In conclusion, primary cardiac angiosarcoma can occur within families even in advanced age, including the seventh decade and beyond. This challenges the conventional belief that hereditary cases occur in younger individuals.

## Data Availability

The data underlying this article will be shared on reasonable request to the corresponding author.
